# *Microbial* Biotechnology: evolution of your premier journal

**DOI:** 10.1111/1751-7915.12009

**Published:** 2012-12-18

**Authors:** Ken Timmis, Juan Luis Ramos, Marty Rosenberg, Willy Verstraete, Willem de Vos

Dear Readers,

*Microbial Biotechnology* (*MBT*) is undergoing a number of interesting changes that we would like to report.

The first is its transition to an author-pays open access model from 2013. We believe open access will offer the journal a number of advantages, including the flexibility to grow without the constraints of a page budget, and substantial increases in exposure, with all articles being made freely available via Wiley Online Library and PubMed Central. *MBT*'s transition to open access will significantly benefit both authors and readers, and hence the field of biotechnology as a whole. The Journal will continue to be editorially independent and its editorial team will continue to apply ever increasing standards of peer review, thus ensuring that the journal continues to be the high quality publication we know and value.

Another is that *MBT* was assigned its first Impact Factor at the end of June and, since that time, has experienced a significant increase in submissions. As has been the case with its sister journals, *Environmental Microbiology* and *Environmental Microbiology Reports*, this rise in submissions will translate into an Editor-implemented rise in quality–originality threshold for acceptance, which in turn will positively influence subsequent IFs. Given the steady increase in exciting science we receive, we are confident that *MBT* will experience healthy increases in IF over the coming years.

An important characteristic of *MBT* is the strength of its Editorial team – its Editors, Editorial Board and army of *ad hoc* reviewers, composed of leaders in the different sectors of microbial biotechnology, leaders both in the sense of representing the field and in pioneering the way forward. It is these dedicated scientists who set the benchmark for the field and for the papers that are published in *MBT*. They do this by providing constructive, critical reviews on submissions and by submitting their own work, and thereby ensure that *MBT* publishes some of the best research from high profile groups. The Editors are deeply indebted to the dedicated selfless support of its Editorial Board and *ad hoc* reviewers: thank you all for your tremendous support!

The declared goal of *MBT* is to promote the field of applied microbiology, *inter alia* by stimulating young researchers to carry out original research on novel topics at the interfaces of the various disciplines that impact on biotechnology. It does so not only by showcasing important developments by leading groups, but also by profiling topics and new developments the Editors consider will lead to ground-breaking discoveries. One means it employs to do this is to publish Special and Themed Issues on such topics, edited by top researchers in the field. Exciting recent ones have been on Microbial Vaccines and Immunomodulators (http://onlinelibrary.wiley.com/doi/10.1111/mbt.2012.5.issue-2/issuetoc) edited by Carlos Guzman, Ennio de Gregorio, Jan ter Meulen and Martin Friede, and Microbial Resource Mangement (http://onlinelibrary.wiley.com/doi/10.1111/mbt.2012.5.issue-3/issuetoc), edited by Nico Boon and Willy Verstraete. Another means is the Web Alert of Larry Wackett which assembles the best websites for relevant diverse aspects of applied microbiology. A third is the biannual original, sometimes provocative, Crystal Ball series, in which international experts speculate on the key new developments/theories/paradigms that will drive discoveries in the field over the following years. This feature, which appears in the first issue of the year, always creates considerable interest and, in some cases, amusement. Take a look at the 2013 CB in this issue.

And finally: check out below the most downloaded and most cited papers in *MBT* (see the *MBT* website for latest updates) to see who is publishing what in hot interest-generating biotechnology.

**Top downloaded articles**

**Marine genomics: at the interface of marine microbial ecology and biodiscovery**

Karla B. Heidelberg, Jack A. Gilbert, Ian Joint

**Bacterial persistence increases as environmental fitness decreases**

Seok Hoon Hong, Xiaoxue Wang, Hazel F. O'Connor, Michael J. Benedik, Thomas K. Wood

**Strategies for discovery and improvement of enzyme function: state of the art and opportunities**

Praveen Kaul, Yasuhisa Asano

**Natural products for cancer chemotherapy**

Arnold L. Demain and Preeti Vaishnav

**Anaerobic benzene degradation by bacteria**

Carsten Vogt, Sabine Kleinsteuber, Hans-Hermann Richnow

**Top cited articles**

**Metabolic engineering to enhance bacterial hydrogen production**

Maeda, T; Sanchez-Torres, V; Wood, TK

**Identification of furfural as a key toxin in lignocellulosic hydrolysates and evolution of a tolerant yeast strain**

Heer, D; Sauer, U

**Microbial degradation of lignin: how a bulky recalcitrant polymer is efficiently recycled in nature and how we can take advantage of this**

Ruiz-Duenas, FJ; Martinez, AT

**Microbial reporters of metal bioavailability**

Magrisso, S; Erel, Y; Belkin, S

**Indole and 7-hydroxyindole diminish Pseudomonas aeruginosa virulence**

Lee, J; Attila, C; Cirillo, SLG; Cirillo, JD; Wood, TK

So, submit your best work to *MBT*, participate in its upward march to become the flagship of microbial biotechnology research, and your paper may be in one of these lists in future!

